# Exploring the Use of Digital Educational Tools for Sexual and Reproductive Health in Sub-Saharan Africa: Systematic Review

**DOI:** 10.2196/63309

**Published:** 2025-02-26

**Authors:** Ramatu Hajia Abdul Hamid Alhassan, Catherine L Haggerty, Abimbola Fapohunda, Nabeeha Jabir Affan, Martina Anto-Ocrah

**Affiliations:** 1 Department of Epidemiology University of Pittsburgh School of Public Health Pittsburgh, PA United States; 2 Department of Behavioral and Community Health Sciences University of Pittsburgh School of Public Health Pittsburgh, PA United States; 3 Department of Medicine Division of General Internal Medicine University of Pittsburgh School of Medicine Pittsburgh, PA United States

**Keywords:** digital health, adolescents, Africa, sexual health, reproductive health, human-centered design

## Abstract

**Background:**

Adolescents, particularly those in Sub-Saharan Africa, experience major challenges in getting accurate and comprehensive sexual and reproductive health (SRH) information because of sociocultural norms, stigma, and limited SRH educational resources. Digital educational tools, leveraging the widespread use of mobile phones and internet connectivity, present a promising avenue to overcome these barriers and enhance SRH education among adolescents in Sub-Saharan Africa.

**Objective:**

We conducted a systematic review to describe (1) the geographic and demographic distributions (designated objectives 1a and 1b, respectively, given their interrelatedness) and (2) the types and relevant impacts of digital educational tools (objective 2).

**Methods:**

We followed the PRISMA (Preferred Reporting Items for Systematic Reviews and Meta-Analyses) guidelines, using databases, such as Ovid-MEDLINE, Google Scholar, PubMed, and ERIC, to conduct literature searches. The selection criteria focused on studies that specifically addressed digital educational tools used to assess or deliver SRH education, their implementation, and their effectiveness among the adolescent population in Sub-Saharan Africa. We used the JBI critical appraisal tools for the quality assessment of papers included in the review.

**Results:**

The review identified 22 studies across Sub-Saharan Africa that met the inclusion criteria. The 22 studies spanned populations in West, Central, East, and South Africa, with an emphasis on youth and adolescents aged 10-24 years, reflecting the critical importance of reaching these age groups with effective, accessible, and engaging health education (objectives 1a and 1b). There was a diverse range of digital tools used, including social media platforms, mobile apps, and gamified learning experiences, for a broad age range of adolescent youth. These methods were generally successful in engaging adolescents by providing them with accessible and relevant SRH information (objective 2). However, challenges, such as the digital divide, the cultural sensitivity of the material, and the necessity for a thorough examination of the long-term influence of these tools on behavior modification, were noted.

**Conclusions:**

Digital educational tools provide great potential to improve SRH education among adolescents in Sub-Saharan Africa. These technologies can help enhance relevant health outcomes and accessibility by delivering information that is easy to understand, interesting, and tailored to their needs. Future research should focus on addressing the identified challenges, including bridging the digital divide, ensuring cultural and contextual relevance of content, and assessing the long-term impact of digital SRH education on adolescent behavior and health outcomes. Policymakers and educators are encouraged to integrate digital tools into SRH educational strategies that target adolescents in order to improve the SRH of this age group and contribute to improving public health in Sub-Saharan Africa.

## Introduction

According to the World Health Organization, there are more than 1.2 billion adolescents aged 10 to 19 years with sexual and reproductive health (SRH) problems, such as sexually transmitted infections (STIs) and unplanned pregnancies, many of whom face significant barriers to accessing SRH-related education. With the increasing rates of SRH problems and mental health issues among adolescents, there is a pressing global need for effective educational tools that address the SRH needs of adolescents [1,2]. There are global disparities in adolescents’ access to SRH education, with those in low- and middle-income countries (LMICs) facing the greatest barriers to accessing knowledge and resources due to sociocultural norms, beliefs, practices, and stigma, and yet, these parts of the world have the greatest burden of poor adolescent SRH outcomes [3]. For example, over 80% of all adolescents living with HIV are in Sub-Saharan Africa [4], which also has the highest prevalence of adolescent pregnancy globally [5]. Melesse et al [6] conducted a review of demographic and health survey data from 33 African countries and reported that despite improvements in SRH among adolescents in Sub-Saharan Africa, young people in this part of the world continue to have disproportionately high rates of unwanted pregnancies and STIs (including HIV/AIDS). These persistently high rates of poor health outcomes have been attributed, at least in part, to limited access to SRH education, more formally referred to as comprehensive sexuality education, in many Sub-Saharan African countries [7]. Challenges impacting access to sex education in Sub-Saharan Africa include cultural and societal norms that serve as barriers to youths’ access to information, especially taboos regarding the open discussion of topics such as family planning, menstruation, and contraception [8]. Additionally, comprehensive sexuality education programs have been constrained by inconsistent funding and poor accountability [9]. It is crucial to promote SRH education to combat the challenges faced by adolescents and foster a more inclusive and equitable society as these young people transition into adulthood. Additionally, universal access to SRH is an essential component of sustainable development goals [10].

Digital educational tools have the potential to effectively provide SRH education for adolescents in Sub-Saharan Africa. Such technologies use the widespread availability of mobile phones and internet infrastructure to innovatively address the unique challenges faced by the youth in accessing current information and resources [11,12]. The use of social media platforms, websites, games, and mobile apps has become prevalent in digital education. These tools can provide instructional content with the potential to stimulate teenagers’ interest in learning and promote favorable attitudes and knowledge that may lead to desirable SRH outcomes. The integration of digital tools into SRH education offers a promising avenue to overcome health disparities [13,14]. Mobile health (mHealth), which is the practice of medicine and public health that uses mobile devices, has exploded over the last few years, with mobile phone penetration reaching 90% in some LMICs [15]. The increased usage of mobile phones among younger populations in LMICs presents a valuable opportunity to use mHealth, for example, as a tool to overcome barriers to accessing SRH information and service [16]. Digital technology has the potential to close the information gap for adolescents in LMICs, ensuring equitable access to essential health knowledge for all adolescents irrespective of their geographical location or socioeconomic status.

We have conducted a systematic review to describe: (1) the geographic and demographic distribution (designated objectives 1a and 1b, respectively, given their interrelatedness) and (2) the types and formats of digital educational tools available for delivering SRH education to adolescents in Sub-Saharan Africa and, as appropriate, the impacts of these digital innovations on target populations (objective 2). A systematic review is appropriate for this work as it allows us to comprehensively synthesize the current state of the literature on such an underresearched topic, identify gaps in knowledge, and provide insights on moving the field forward [17]. The public health significance of this review lies in its potential to inform policy and practice by highlighting successful digital interventions that can enhance SRH education for adolescents. Given the increasing prevalence of internet and mobile device usage in Sub-Saharan Africa, identifying effective educational strategies is crucial for addressing the high rates of unintended pregnancies, STIs, and other reproductive health challenges faced by young people in the region. Ultimately, our findings can be used to support the development of targeted evidence-based digital interventions that can significantly improve health outcomes for adolescents in this part of the world.

## Methods

### Definitions of Digital Tools and Adolescents

We have defined the terms “digital tools” and “adolescents” as presented below.

#### Digital Tools

Digital tools are technological resources or applications used in the provision of information, support, and resources related to SRH. These tools use digital technology to enhance access, outreach, and effectiveness in the delivery of SRH education and services. They are available through a variety of digital platforms, such as websites, mobile apps, and social media platforms [11]. The use of such tools has become increasingly prevalent in recent years as they offer a convenient and efficient means of accessing information and resources related to SRH. These tools have gained considerable popularity owing to their ease of use and convenience in terms of obtaining information related to SRH.

#### Adolescents

Adolescents, who are individuals in the intermediate stage of development between childhood and adulthood, commonly referred to as teenagers or young adults, experience substantial transformations in physical structure, thinking, interpersonal relationships, and emotions [18]. As adolescents face major educational and health challenges, especially in the context of SRH-related information, our review is focused on this age group. We used the World Health Organization’s definition of “adolescents” as individuals aged 10 to 19 years, “youth” as those aged 15 to 24 years, and “young people” as those aged 10 to 24 years [19]. Therefore, if any studies included these age groups, we erred on the side of caution and included them to ensure we were being as comprehensive as possible in capturing the breadth and scope of the research in this field.

### Database and Search Dates

This systematic review adhered to the PRISMA (Preferred Reporting Items for Systematic Reviews and Meta-Analyses) criteria [20] to ensure that all relevant primary materials match the review requirements [21]. Additionally, we used the JBI critical appraisal tools for the quality assessment of all papers included in the review [22] (Multimedia Appendix 1). A systematic search of peer-reviewed articles was conducted in November 2023 using Ovid-MEDLINE, with the assistance of a University of Pittsburgh Health Sciences Library System (HSLS) professional, to identify original research articles published between 2013 and 2024. The first Ovid-MEDLINE search was developed using a mix of Medical Subject Heading (MeSH) phrases, keywords, and Boolean operators (eg, “adolescent* or teen* or youth”; “digital health education/ or health promotion/ or sex education/”; “Africa or Angola or Benin or Botswana or Burkina Faso or Burundi or Cabo Verde or Cameroon or Central African Republic or Chad or Comoros or Congo or Cote d'Ivoire or Djibouti or Eritrea or Eswatini or Ethiopia or Gabon or Gambia or Ghana or Accra or Kumasi or Tamale or Sekondi-Takoradi or Guinea or Kenya or Lesotho or Liberia or Madagascar or Malawi or Mali or Mauritania or Mozambique or Namibia or Niger or Nigeria or Rwanda or Sao Tome or Principe or Senegal or Seychelles or Sierra Leone or Somalia or Sudan or Tanzania or Togo or Uganda or Zambia or Zimbabwe.ti,ab,kf.”) (Multimedia Appendix 1). The search results were managed and tracked in SciWheel.

### Study Selection

We restricted the search to studies conducted in humans and written in the English language. Additional articles were identified by cross-referencing bibliography lists. In alignment with the PRISMA standards, the methodology encompasses 4 phases: identification of sources, assessment of eligibility, detailed screening, and final selection for inclusion [21]. Initially, potential sources were selected by examining their titles and abstracts, ensuring relevance to the research question. This meticulous process facilitated the systematic evaluation and filtering of records, governed by rigorously defined inclusion and exclusion criteria. Articles that advanced beyond the preliminary screening phase were subjected to a comprehensive review of their full texts to determine their eligibility for inclusion in the systematic review. Articles were first reviewed by the first author (RHAHA) in collaboration with the co-author NJA and under the supervision of the second author (CLH) to ensure they met the eligibility criteria. The JBI critical appraisal tools for the quality assessment of all papers included in the review were further used to ensure the papers met the necessary standards for research and publication (internally valid, appropriately randomized, participants appropriately consented, etc). The eligibility criteria are presented in Textbox 1.

**Eligibility criteria.**

**Inclusion criteria**

- Region: Focuses on the Sub-Saharan African region or has significant relevance to Sub-Saharan African populations
- Target population: Adolescents and youth
- Area of focus: Addresses sexual and reproductive health, including sexually transmitted infection education, mental health, sexuality, and positive sexuality
- Mode of delivery: Digital tools, including apps, websites, gaming, e-learning modules, social media, etc
- Timeline: Published within the last 10 years (2013-2024) to ensure relevance
- Language: In English to ensure comprehension and interpretation by research teams who are primarily English speaking

**Exclusion criteria**

- Region: Excluded Sub-Saharan African populations or did not have a Sub-Saharan Africa focus
- Target population: Outside of the age range of adolescents and young people
- Area of focus: Does not specifically address sexual and reproductive health
- Mode of delivery: Excludes digital tools
- Timeline: Outside of the specified dates
- Language: Not in English

## Results

### Included Studies

**Figure 1:**
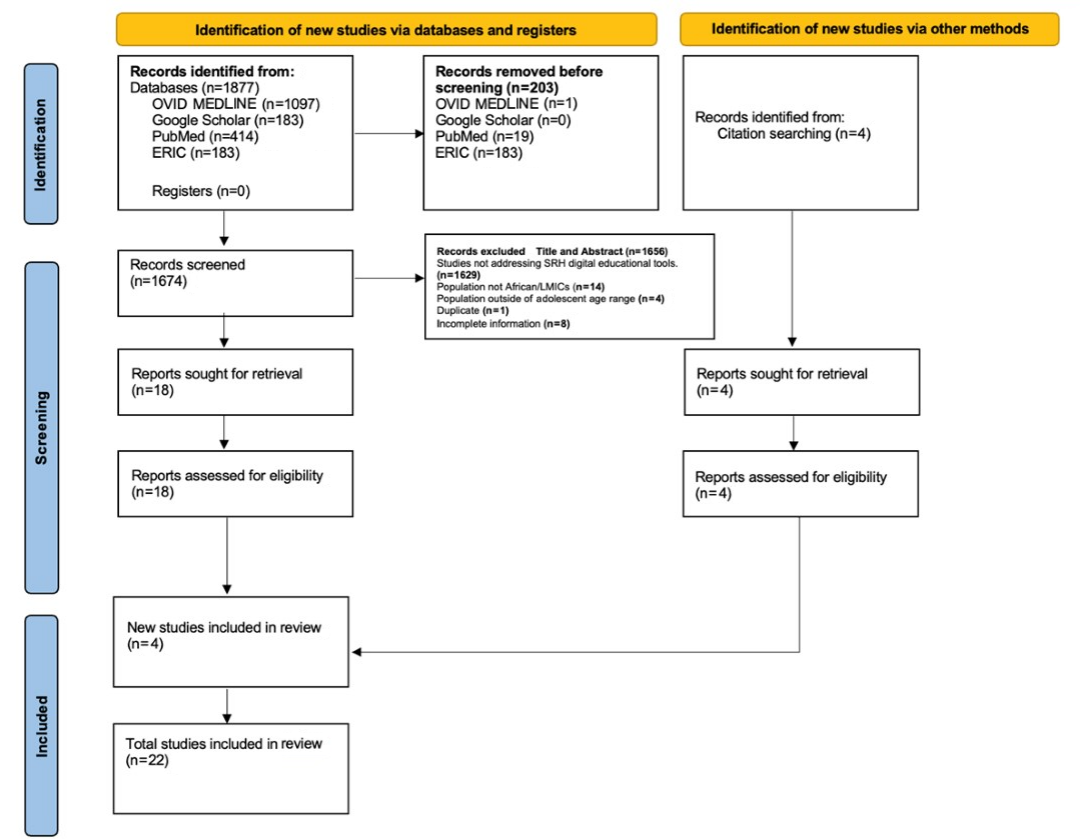
Flowchart for our systematic review of digital educational tools for adolescent sexual and reproductive health (SRH) in Sub-Saharan Africa. LMIC: low- and middle-income country.

Figure 1 outlines the systematic and comprehensive search and selection process employed in this systematic review, focusing on digital educational tools for adolescent SRH in Sub-Saharan Africa. Initially, a total of 1877 records were identified through meticulous searches across major databases, including OVID-MEDLINE, Google Scholar, PubMed, and ERIC. An additional 4 records were identified through citation searching, augmenting the pool of potential studies. Before screening, 203 records were removed due to duplication, leaving 1674 records for detailed screening. The screening process, grounded in rigorously defined inclusion and exclusion criteria, led to the exclusion of 1656 records for reasons such as not directly addressing SRH digital educational tools, not focusing on the Sub-Saharan African region or LMIC context, targeting populations outside the adolescent age range and young people, and duplications. After the exclusion process, 18 reports were retrieved and assessed for eligibility, and 4 additional studies were identified and included in the review alongside the initially identified studies, bringing the total number of studies included in the review to 22 (Figure 1; Table 1) [22].

**Table 1:** Summary of studies on the availability of digital sexual and reproductive health education for adolescents in Sub-Saharan Africa.

Study (authors and year)	Study population/region/country	Study design	Digital tool type	Findings
Dulli et al [23], 2020	349 youth aged 15-24 years from Nigeria	Randomized controlled trial. Participants were randomized into 2 groups: those receiving the SMART Connections intervention and those receiving standard care services.	Social media	Primary results: The intervention did not significantly improve retention (50% vs 45% retention in treatment vs control groups) or social support but did significantly improve HIV-related knowledge (*t*=–2.96; *P*=.003).
Fakoya et al [24], 2022	111 adolescents aged 15-19 years from Nigeria, Tanzania, and Ethiopia	Application of youth-led participatory action research (PAR) approaches within human-centered design (HCD)	Youth-engaged version of HCD as part of the Adolescents 360 initiative	Primary results: Successful engagement of youth as project partners and action researchers. Identified opportunities to improve program empathy and responsiveness. Challenges in recruiting “extreme users” due to high competencies needed in HCD. Empathy and design standards during prototyping helped in decision-making. Real-world testing of services and products emphasized the importance of continuing youth-adult partnership.
Feroz et al [16], 2021	Young people (adolescents and youth) aged 10-24 years in low- and middle-income countries (LMICs), specifically Sub-Saharan Africa and Asia	Systematic review	Mobile phones used for mHealth interventions aimed at improving sexual and reproductive health (SRH) outcomes	Primarily used for client education and behavior change communication (n=14, 93%), followed by financial transactions and incentives, and data collection and reporting. Studies evaluated the effect mHealth interventions had on access to SRH services (n=9) and SRH outcomes (n=6). mHealth interventions improved access to SRH services and outcomes by addressing barriers such as provider prejudice, stigmatization, discrimination, and issues of privacy and confidentiality. Included decreased technological literacy, inferior network coverage, and lower linguistic competency.
Gonsalves et al [25], 2018	705 young people aged 13-24 years from Kenya and Peru	Protocol of an open, 3-arm, individually randomized trial. Participants will be randomized into one of three arms: (1) intervention arm receiving SRH information (using ARMADILLO) via mobile phones, (2) control arm receiving no intervention, and (3) a third arm with variations by site (Kenya and Peru).	Mobile phone sexual health information	If proven to be effective, interventions like ARMADILLO can bridge an important gap in achieving universal access to SRH information and education for an otherwise difficult-to-reach group. Outcomes will be assessed via questionnaires administered at baseline, intervention end, and 8 weeks after intervention end. Statistical analysis will include comparisons of proportions (chi-square tests) and means (*t*-tests) between arms and difference-in-difference techniques.
Haruna et al [26], 2019	348 secondary school participants (students) aged 11-15 years from 3 schools were recruited to participate in this study from Tanzania	Design-based research (DBR)	Digital gamified learning platform named “My Future Begins Today”	A paired *t*-test revealed a statistically significant improvement in sexual health literacy scores from pretest (mean 26.40, SD 7.29) to posttest (mean 74.12, SD 16.21) (t_347_=52.230; *P*<.000), with an average increase of 47.72 points. A 1-way ANOVA showed no significant differences among the 3 groups’ pretest scores, but significant differences were found in posttest scores (*F*_2,345_=210.43; *P*<.001), with follow-up Tukey post hoc tests indicating better performance by the gamified learning platform group compared to the traditional teaching group. Positive stakeholder and participant feedback on the gamified approach. Effective use of participatory design in developing educational tools.
Nalwanga et al [27], 2021	1086 Kyambogo University students aged 18-30 years from Uganda	Cross-sectional analysis of data from an endline survey of a randomized controlled trial (RCT) and data from use of a mobile phone app over a 6-month period.	Mobile phone app	The mobile phone app demonstrated predominantly positive (responsiveness, nondistracting in-app advertisements, and ease of use) attributes. 86% (n=464) of students who received a recommendation to download the app accepted and downloaded it. Of these students, 81% (n=374) used the app to access SRH information, goods, and services over 6 months. In terms of responsiveness, 55.1% (n=206) of students stated that clinics were responsive (*P*=.11). 75.4% (n=282) said the in-app advertisements were not distracting (*P*=.23). 44.4% (n=166) found that in-app instructions were very easy to use (*P*=.50).
Ippoliti et al [28], 2017	Adolescents and youth aged 10-24 years from Africa (67%), Eurasia (26%), and Latin America (13%)	Global landscape analysis. The authors issued a global call for project resources in 2014 and reviewed the submissions to confirm they met specific inclusion criteria.	Mobile phones (including SMS text messaging and mobile phone apps)	Most projects (n=12, 70%) relied on text messaging to transmit SRH information. The majority of projects were based in Africa (67%), followed by Eurasia (26%) and Latin America (13%). Mobile phones were effectively used to increase the reach of SRH information and services, especially in conservative societies where SRH topics are stigmatized. 70% of the projects relied on text messaging to transmit SRH information. Several projects have been adapted and scaled to other countries, demonstrating the scalability of mHealth interventions.
L’Engle et al [29], 2016	Review targeted adolescents aged 10-24 years and included studies that provided results from mobile phone interventions designed to improve adolescent SRH	Systematic review	Mobile phone interventions, primarily text messaging	Most programs (n=23, 82%) used text messaging. An average of 41% of essential mHealth criteria were met. An average of 82% of methodological reporting criteria were met. Evidence suggests improvements in health promotion campaigns, sexually transmitted infection (STI) screening and follow-up, and medication adherence.
Nolan et al [30], 2020	6000 youth aged 12-19 years across 8 districts in Rwanda	3-arm cluster-randomized. Arms: (1) CyberRwanda self-service, (2) CyberRwanda facilitated, and (3) control schools, which will receive the standard services that are available in the community.	CyberRwanda, a digital health intervention consisting of interactive stories, questions and answers, videos, and an online shop for health products	The study protocol describes the design and intended impact evaluation of the CyberRwanda program using a 3-arm cluster randomized noninferiority trial, but it does not report on the outcomes or effectiveness of the intervention. The primary outcomes are measurements of uptake of a modern method of contraception, initiation of childbearing, and HIV testing, all measured at the participant level. Data analysis will consist of a generalized linear mixed model.
Onukwugha et al [31], 2022	Review includes individuals aged 10-19 years, with consideration for interventions focusing on young people aged 10-24 years in Sub-Saharan Africa	Systematic review	mHealth interventions	mHealth interventions were effective in improving adolescents’ uptake of SRH services across a wide range of services, with the strongest evidence for contraceptive use. Interventions with 2-way interactive functions and more behavior change techniques embedded were more effective. Limited data suggested that interventions were inexpensive, but cost-effectiveness was not evaluated.
Patel et al [32], 2022	Adolescents in LMICs (countries not specified by the authors)	Environmental scan is used to identify current SRH mobile apps available in the iOS App Store and Android Play Store.	Mobile apps	2165 mobile apps were initially identified, with only 8 apps meeting the inclusion criteria and assessed using the Mobile App Rating Scale (MARS) tool. The functionality subdomain scored the highest at 4.6. The information subdomain scored the lowest at 2.5. None of the assessed apps contained information on the MARS items related to the evidence base and goals. “Too Shy to Ask” had the highest individual app mean score of 4.1, while “e-SRHR” scored the lowest at 2.3.
Pfeiffer et al [33], 2014	60 adolescents aged 15-19 years in Dar es Salaam and Mtwara, Southern Tanzania	Mixed methods study	Social media, with a particular focus on Facebook	Adolescents access the internet mainly through mobile phones (68%). Facebook is the most popular internet site among the youth (73%). Adolescents expressed interest in receiving reproductive and sexual health messages through social media, with 92% of respondents saying Facebook should be used. Youth role models, such as music stars and actors, are influential in delivering health messages.
Rogers et al [34], 2019	Adolescents aged 10-19 years living in Zambia	Content analysis of the TuneMe website	Mobile-optimized website (TuneMe)	The TuneMe platform provided extensive information on SRH and HIV. Topics on relationships (21%) as well as sexual rights and sexual citizenship (11%) were also covered, albeit to a lesser extent. Areas, such as pleasure, violence, diversity, and gender, received significantly less attention. Content was presented in culturally relevant contexts but sometimes portrayed mixed or problematic views on gender norms. 89% of the TuneMe content falls within the IPPF Framework components for CSE, with 37% falling in the SRH and HIV primary category. The Gunning Fog Index score indicated that a minimum of an 8th grade reading level would be required to fully understand TuneMe content.
Rokicki et al [35], 2017	756 female adolescents aged 14-24 years enrolled in secondary schools in Accra, Ghana	Cluster randomized controlled trial. Unidirectional intervention (n=12 schools): participants received text messages with reproductive health information; interactive intervention (n=12 schools): participants engaged in text messaging reproductive health quizzes; control (n=14 schools): did not receive reproductive health–related text messages.	Text messaging program	The unidirectional intervention increased reproductive health knowledge by 11 percentage points (95% CI 7-15). The interactive intervention increased knowledge by 24 percentage points (95% CI 19-28) from a control baseline of 26%. No significant changes in reproductive health outcomes overall. Both interventions lowered the odds of self-reported pregnancy for sexually active participants (unidirectional: OR 0.14, 95% CI 0.03-0.71; interactive: OR 0.15, 95% CI 0.03-0.86). Results showed that text-messaging programs may help improve reproductive health knowledge among adolescents.
Soehnchen et al [13], 2023	77 young adults aged 18-35 years in the resource-poor regions of Kenya	Survey based on the Unified Theory of Acceptance and Use of Technology (UTAUT)	Web-based prototype designed to provide essential sexual health information	77 responses were included in the analysis. High acceptance and usability of the digital tool among the target population. Perceived usefulness, attitude toward health care–integrated evidence technology, and usability had significant positive impacts on acceptance and intention to use. Having the resources and knowledge necessary for the usage of a digital tool had a significant negative impact. The chi-square test statistic was 532 (*P*<.001), meaning it was highly significant compared to the original model. A System Usability Scale (SUS) score of 67.3 indicated the tool’s usability as “okay.”
Soehnchen et al [8], 2023	17 young female individuals aged 15-25 years from Kenya	Qualitative research design, with a sample size of 12 pilot phase interviews and 5 expert interviews.	Web-based sexual health education app	Stigmatization around sexual health in Kenya leads to myths and a lack of information. Sexual health education is not part of the Kenyan school curriculum, leading to insufficient knowledge about safe contraception, menstruation, and female genital mutilation. A digital app could support and provide education and information for universal equal access. Barriers to using a digital sexual health education app include conservative cultural background, classic text communication, and social affiliation influence.
Frankline et al [36], 2020	398 female adolescents aged 10-19 years from Cameroon	Single-centered randomized controlled single-blinded trial. Intervention group: received weekly educational 1-way text messages on sexoreproductive health (199 participants); control group: did not receive the text messages (199 participants).	Mobile phone text messaging (SMS)	Significant increase in mean knowledge, attitude, and practice scores from baseline to the end of the study (from 6.03, 4.01, and 3.45 to 7.99, 5.83, and 4.99, respectively). Statistical significance in the overall improvement in adolescents’ perception of sexoreproductive health as a result of the intervention (*F*=15.12; *P*=.02 for knowledge; *F*=60.21; *P*=.001 for attitudes; *F*=57; *P*=.01 for practices). Majority (65.3%) of participants were satisfied with the SMS text messaging service.
Akande et al [37], 2024	1280 in-school adolescents aged 15-17 years from Nigeria	Cluster randomized controlled trial. Intervention group: received the mHealth-based intervention; control group: did not receive the mHealth intervention.	mHealth-based sex education program	Significant improvements in SRH knowledge (*F*=2117.252; *P*<.001) and attitudes (*F*=148.493; *P*<.001) in the intervention group. No significant change in risky sexual behavior scores in the intervention group (*P*=.57). Females had higher odds of having good SRH knowledge (aOR 2.5, 95% CI 1.04-6.13). Males had lower odds of practicing protective sexual behavior (aOR 0.3, 95% CI 0.15-0.55), and higher class levels (SS2: aOR 5.2; SS3: aOR 6.2) were associated with more protective behavior.
Rokicki and Fink [15], 2017	756 female adolescents and young women aged 14-24 years from Ghana	Randomized controlled trial. Unidirectional intervention (text messages with reproductive health information), interactive intervention (engaged adolescents in text messaging reproductive health quizzes), and control (no intervention).	Interactive mobile phone quiz (mHealth intervention)	The mHealth intervention was effective in engaging adolescent girls across sociodemographic strata. Participants showed increased health knowledge regarding SRH. Knowledge scores increased from 26% to 32% in the control group, 30% to 45% in the unidirectional group, and 31% to 60% in the interactive group when looking from baseline to the 3-month follow-up. The interactive intervention was more effective than the unidirectional intervention, increasing knowledge scores by 13 percentage points (95% CI 8-18). The intervention reached adolescents who may be at higher risk of poor SRH outcomes.
Haruna et al [38], 2018	120 lower secondary school students aged 11-15 years from Tanzania	Randomized controlled trial. Groups: (1) game-based learning (GBL), (2) gamification, (3) control group (traditional teaching).	GBL and gamification platforms	The Adolescent Sexual Health Literacy Test (ASHLT) showed a significant increase in scores from pretest (mean 29.26, SD 8.689) to posttest (mean 70.36, SD 18.201) (t_119_=–23.787; *P*=.001). GBL and gamification methods significantly improved students’ motivation, attitude, knowledge, and engagement (MAKE) compared to traditional teaching methods, as evidenced by higher scores in these areas and supported by positive feedback from focus group discussions. Pairwise comparisons revealed significant differences between traditional and experimental methods, with no substantial differences between GBL and gamification, indicating that both experimental approaches were more effective in enhancing sexual health education.
Alhassan et al [39], 2019	250 adolescents and young people aged 18-24 years at the University of Ghana, Legon in Accra, Ghana	Cross-sectional analytical study	Mobile phones (including smartphones)	99% of participants owned mobile phones, with 58% being smartphone users. Male young adults and young adults who owned a smartphone were more likely to use mobile phones for STI education and prevention (*P*=.000 and *P*=.01, respectively). The study suggests high mobile phone penetration among young adults, with a belief in the efficacy of mobile phone programs for STI education and prevention.
Ippoliti et al [40], 2021	Adolescents aged 12-19 years from Rwanda	HCD approach	CyberRwanda, a web-based digital health platform	Over 1000 Rwandan youth, caregivers, teachers, health care providers, and government stakeholders engaged. Revealed participants’ beliefs, behavioral preferences, and experiences related to family planning and reproductive health. Designed for urban and periurban young people and pharmacy staff. Evaluations will be conducted across 60 schools and 9 youth centers in 8 districts in Rwanda (n=6082 at baseline).

### Objectives 1a and 1b: Geographic and Demographic Focus

**Figure 2:**
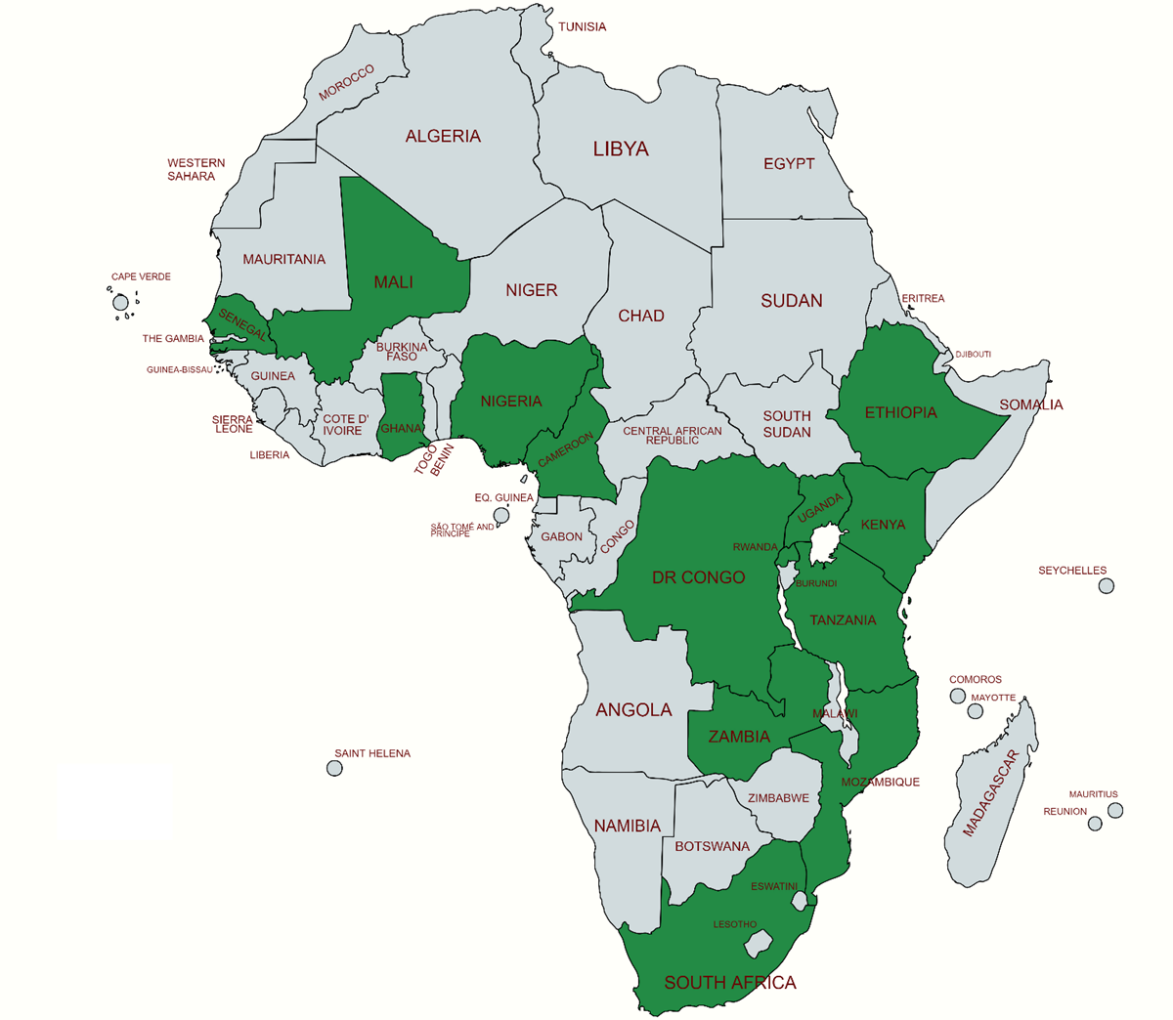
Map of Africa showing the countries included in the reviewed studies (green).

As shown in Figure 2, the geographical scope of research on digital interventions for SRH education spans diverse settings in Sub-Saharan Africa, each offering unique insights into the challenges and opportunities inherent in deploying technology-based solutions within various cultural and infrastructural contexts. The studies span populations in West, Central, East, and South Africa. This geographical diversity is not merely a backdrop for these studies but a critical factor that shapes the design, implementation, and impact of digital health interventions across the continent; it underscores the importance of contextually adapted solutions. As depicted in Table 1, the studies included youth and adolescents of various ages, integrating variations of human-centered designs (HCDs) that resonated with their lived experiences and preferences. In Nigeria, Dulli et al [23], Fakoya et al [24], and Akande et al [37] highlighted the country’s engagement with digital platforms to address SRH among youth and adolescents. Feroz et al [16] and Ippoliti and L’Engle [28] also included studies from Nigeria. Nigeria, with its vast population and significant digital penetration, represents a critical context for understanding how social media and participatory design can be harnessed to meet young people’s health education needs. The focus on Nigeria underscores the potential of digital tools to transcend traditional barriers to health education, leveraging the widespread use of social media and mobile phones among the youth. Besides Nigeria, this study included some other countries in West Africa, such as Ghana [16,31,35,39], Senegal, and Mali [28].

The reviewed studies extend beyond West Africa to include Kenya, as illustrated by Gonsalves et al [25], who explored the impact of mobile phone–based sexual health information among young people, as well as Soehnchen et al [13], who assessed acceptance of a digital tool for delivering sexual health education. Some other authors, namely Feroz et al [16], Ippoliti and L’Engle [28], Onukwugha et al [31], and Soehnchen et al [8], also included studies that were performed in Kenya in their reviews. Kenya’s inclusion in this body of research is indicative of its role as a hub for technological innovation and digital health initiatives in East Africa. Nalwanga et al [27] performed a cross-sectional analysis to assess the use of a mobile phone app by university students in Uganda, another East African country. Haruna et al [26] focused on secondary school participants in Tanzania, using a gamified learning platform to engage students in SRH education. This emphasis on educational settings highlights the potential of integrating digital tools into formal education systems, showing how gamification can enhance learning outcomes and engagement among school-aged youth. Data from Tanzania, Rwanda, and Ethiopia, which are also East African countries, were included in studies by Fakoya et al [24], Feroz et al [16], Haruna et al [26,38], Ippoliti and L’Engle [28], Ippoliti et al [40], Onukwugha et al [31], Nolan et al [30], and Pfeiffer et al [33]. The cross-regional approach of Gonsalves et al [25], encompassing participants from both Kenya and Peru, further emphasizes the global relevance of digital health interventions and the importance of considering diverse cultural and infrastructural landscapes in their design and deployment.

This review also included some countries in southern Africa, including South Africa, Mozambique, and Zambia. For example, Rogers et al [34] performed a content analysis of TuneMe, a mobile-optimized website that offers extensive information on SRH and HIV. Importantly, the content was presented in culturally relevant contexts. In addition to that study, Feroz et al [16], Ippoliti and L’Engle [28], and Onukwugha et al [31] included data from southern African countries in their systematic reviews.

Democratic Republic of Congo [16] and Cameroon [36] were the 2 Central African countries included in this review. Collectively, these studies paint a picture of a continent actively engaging with digital technologies to overcome the challenges in delivering SRH education. The wide geographical reach of this research, ranging from the populous country of Nigeria to the tech-savvy environments of Kenya, reflects the varied landscapes in which these digital interventions are implemented. Each setting provides unique insights into the interplay between cultural norms, technological accessibility, and health education needs, contributing to a richer understanding of how digital tools can be adapted and scaled across the Sub-Saharan African context.

### Target Demographics

Among the studies reviewed, there was a notable emphasis on youth and adolescents, spanning ages 10 to 24 years, which reflected the critical importance of reaching these age groups with effective, accessible, and engaging health education. Although the focus of this study is the adolescent age group, they are often included in broader age groups, such as young people and youth, as shown in Table 1. For instance, Dulli et al [23] concentrated on youth aged 15 to 24 years in Nigeria. The authors performed a randomized controlled trial using social media as a platform to deliver sexual health interventions (SMART Connections). Similar populations were targeted by Gonsalves et al [25] in Kenya and Rokicki et al [35] in Ghana. Similarly, Fakoya et al [24] engaged adolescents aged 15 to 19 years from Nigeria, Tanzania, and Ethiopia, incorporating youth-led participatory action research with an HCD to create interventions that resonate with the lived experiences and preferences of young individuals. The authors directly engaged the target demographic in the study design process. In Rwanda, Nolan et al [30] analyzed data from 6000 youth aged 12 to 19 years in a cluster-randomized trial and Rogers et al [34] studied a similar age group (10 to 19 years) in Zambia.

Further broadening the demographic spectrum, Feroz et al [16] extended their review to young people aged 10 to 24 years in Sub-Saharan Africa and Asia. The Sub-Saharan African countries in that study included Tanzania, Nigeria, Ghana, Uganda, and Kenya. The authors performed a comprehensive review of mHealth interventions, identified a wide range of options, and elucidated their applications as well as barriers and facilitators for adoption.

The inclusive age range spanning from early adolescence to young adulthood acknowledges the diverse needs and challenges faced by individuals as they navigate through different stages of the early life course, emphasizing the importance of tailoring interventions to meet these varying requirements. The more expansive approach of Gonsalves et al [25], by including young people aged 13 to 24 years in Kenya and Peru, illustrates the global relevance of digital sexual health interventions and the need to consider cross-cultural applicability and customization in their design and implementation. This cross-regional study underscores the universal challenges related to sexual health education, while acknowledging the specific nuances that different cultural contexts bring to the fore. Finally, Haruna et al [26] involved secondary school participants from Dar es Salaam, Tanzania in the development of a digital gamified learning platform. This engagement not only emphasizes the educational context as a critical setting for sexual health education but also showcases the potential of gamification as a strategy to enhance learning outcomes and retention among school-aged youth.

Collectively, the studies reflect a strategic and inclusive approach, which is aimed at bridging gaps in access and engagement. By examining the implementation and impact of these digital tools across various African settings, researchers and practitioners can gain valuable insights into the factors that drive the success and scalability of health education interventions, thus paving the way for more inclusive and effective public health strategies.

### Objective 2: Digital Tool Types and Effectiveness

The landscape of digital tools deployed for SRH education in various African contexts not only signifies a shift toward more accessible and engaging formats for health education but also reflects the adaptability of health practitioners and researchers to leverage technology to meet the needs of target populations.

With regard to design, Fakoya et al [24] introduced a youth-engaged version of HCD, a problem-solving participatory process that centers the needs, perspectives, and experiences of target populations, when developing solutions to complex challenges. The authors used HCD as part of Adolescents 360, a transdisciplinary initiative to increase the use of modern contraception among 15- to 19-year-old girls in Nigeria, Ethiopia, and Tanzania. The youth-led participatory approach involved 111 “youth designers” trained in HCD methods to inform the design and implementation of the interventions. The authors had successfully engaged youth as project partners and action researchers involved in an iterative cycle of program research, design, and evaluation. However, they noted challenges in recruiting “extreme users” due to the high competencies needed in HCD and emphasized the need for real-world testing (not just prototyping to enhance adolescents’ experiences) and the importance of planned and dedicated financial and human resources. An HCD approach was also deployed in Rwanda by Ippoliti et al [40] for their web-based digital health platform CyberRwanda. The involvement of not only youth but also caregivers, teachers, health care providers, and government stakeholders revealed participants’ beliefs, behavioral preferences, and experiences related to family planning and reproductive health. The authors noted that the HCD process, although iterative, resulted in significant pivots to the design and implementation of the digital platform and resulted in a superior digital health intervention with and for Rwandan youth.

For deployment and implementation, the ubiquity and accessibility of mobile phones make them an ideal platform for reaching a wide audience, particularly in regions where mobile technology may be more readily available than traditional health care services. In fact, Alhassan et al [39] report that 99% of 250 surveyed adolescents and young people aged 18 to 24 years owned mobile phones and 58% were smartphone users. Young male adults and those who owned a smartphone were more likely to use mobile phones for STI education and prevention. In their systematic review of the use of mobile phone technology in LMICs for mHealth SRH interventions, Feroz et al [16] noted that most of the included mHealth intervention studies (n=14, 93%) focused on behavioral change and patient education using a variety of modalities, including SMS text messaging, video clips, images, and voice communication. mHealth tools improved young people’s SRH knowledge, ensured safer sexual behavior, maximized reach and access to family planning information, and improved several HIV outcomes. Barriers to mHealth uptake for SRH included high cost of service, infrastructural or network quality, request for sociodemographic information that could break anonymity, and sociocultural beliefs and norms. Ippoliti and L’Engle [28] also echoed these findings in their global landscape analysis of the use of mobile phones for SRH content among adolescents and youth aged 10 to 24 years in Africa, Eurasia, and Latin America. The authors reported that mobile phones (particularly text messaging) were effectively used to increase the reach of SRH information and services, especially in conservative societies where SRH topics are stigmatized. Nalwanga et al [27] assessed the use of a mobile phone app to increase access to SRH information, goods, and services among university students in Uganda and reported high use of the frequently asked questions portal (71%) and high product use by both sexes (condoms for males [77% use]; sanitary pads for females [94% use]), and the most popularly accessed service was HIV testing and counseling (60% use). Participants appreciated the responsiveness, nondistracting in-app advertisements, and “ease of use” attributes of the mobile phone app. One interesting aspect of this study was the inclusion of a modest co-payment (paid by users), which, interestingly, was not a barrier to usage. Contrary to what has been reported by others, the youth in this study did not find the co-pay to be cost prohibitive. In fact, its incorporation ensured the utility of client payments in future iterations of the app after the pilot period. In a cluster randomized controlled trial of 756 adolescent female individuals aged 14 to 24 years enrolled in secondary schools in Accra, Ghana, Rokicki et al [35] compared the following 3 arms: unidirectional intervention (n=12 schools), where participants received text messages with reproductive health information; interactive intervention (n=12 schools), where participants engaged in text messaging reproductive health quizzes; and control (n=14 schools), where students did not receive reproductive health messaging. They found that the unidirectional intervention increased reproductive health knowledge by 11 percentage points (95% CI 7-15); however, the inclusion of quizzes in the interactive intervention increased knowledge by 24 percentage points (95% CI 19-28) from a control baseline of 26%. Both interventions lowered the odds of self-reported pregnancy for sexually active participants (unidirectional: OR 0.14, 95% CI 0.03-0.71; interactive: OR 0.15, 95% CI 0.03-0.86). These studies highlight the versatility of mobile phones in delivering tailored health education and interventions ranging from SMS text messaging–based information dissemination to more interactive app-based learning experiences and quizzes to reinforce knowledge retention and behavioral change. They can act as a one-stop shop, offering not only educational and clinical counseling services but also condoms, contraceptives, and menstrual products, which give users a holistic experience that can be anonymized to ensure privacy and protection.

The digital gamified learning platform developed in Tanzania by Haruna et al [26,38] represents an innovative leap in engaging and educating secondary school participants in the African setting. The authors used game-based and participatory designs to evaluate the ability of the game to motivate students, improve their attitudes, increase their acquisition of knowledge, and engage them in learning. A paired *t*-test revealed a statistically significant improvement in sexual health literacy scores from pretest (mean 26.40, SD 7.29) to posttest (mean 74.12, SD 16.21) (t_347_=52.230; *P*<.000), with an average increase of 47.72 points. Additional analyses with ANOVA testing showed no significant differences in pretest scores among the 3 groups, but significant differences were found in posttest scores (*F*_2,345_=210.43; *P*<.001), with follow-up Tukey post-hoc tests indicating better performance in the gamified learning platform groups than in the traditional teaching group. These findings suggest that gamified learning platforms can enhance motivation, attitude, knowledge acquisition, and engagement in sexual health education more effectively than traditional methods [26].

The research team took the intervention further in a 3-armed randomized clinical trial to compare the effectiveness of game-based learning and gamification to a control approach (traditional teaching method) [38]. The results showed that the average posttest scores were significantly higher for game-based learning (mean 79.94, SD 11.169) and gamification (mean 79.23, SD 9.186) than for the control approach (mean 51.93, SD 18.705) (*F*_2,117_=54.75; *P*=.001). Additionally, statistically significant differences (*P*≤.05) were found for the constructs of motivation, attitude, knowledge, and engagement (MAKE) for the 2 intervention groups compared to the control arm. Gamification not only enhances learning outcomes but also introduces an element of fun and interaction that can significantly increase participant engagement and knowledge retention [38]. Such gamified platforms offer promising avenues for making health education more appealing and effective for younger people.

Web-based platforms may eliminate the costs associated with the implementation of gamified learning platforms. In a content analysis of the TuneMe website, designed to deliver SRH information to Zambian adolescents aged 10 to 19 years, Rogers et al [34] reported that the website comprehensively covered topics of reproductive anatomy and STIs, including HIV, as well as relationships and sexual rights. However, the challenge of presenting SRH education in culturally relevant contexts was apparent when topics of sexual pleasure and gendered norms, behaviors, expectations, and violence were introduced, demonstrating nuanced beliefs around sexual rights.

Finally, social media emerged as a significant platform in the digital toolbox, with Dulli et al [23] exploring its utility among youth aged 15 to 24 years in Nigeria. The authors performed a randomized controlled trial using social media to deliver sexual health interventions for youth living with HIV (SMART Connections). The design and content of SMART Connections were as informed through workshops conducted with stakeholders and youth living with HIV. The content was delivered anonymously through Facebook over approximately 22 weeks to the intervention and control groups, with daily activities to promote engagement. The primary outcome was retention in HIV treatment, while secondary outcomes included antiretroviral therapy (ART) adherence, HIV knowledge, and social support. Although the intervention did not significantly improve the primary outcome of retention (50% and 45% retention in the treatment and control groups, respectively), there was a significant improvement in HIV-related knowledge for the SMART Connections group compared to controls (*t*=–2.96; *P*=.003). Intervention group participants overwhelmingly reported that the intervention was useful, that they enjoyed taking part, and that they would recommend it to peers. Similarly, Pfeiffer et al [33] found that Facebook was the most used social media platform for sexual health promotion in Southern Tanzania for youth aged 15 to 19 years. Adolescents were most interested in SRH messages through humorous posts, links, and clips, as well as through youth role models like music stars and actors in a manner that reflected up-to-date trends of modern youth culture. Both studies highlighted the integral role of social media in the daily lives of youth, positioning it as a critical channel for delivering sexual health education.

Collectively, these digital tools, ranging from social media and participatory design to mobile phone apps and gamification, reflect a dynamic and multifaceted digital ecosystem for SRH education. Each tool, with its unique features and applications, contributes to a broader strategy aimed at enhancing the accessibility, engagement, and effectiveness of health interventions in different African settings. These diversified digital tools not only address the varied needs and preferences of target demographics but also showcase the potential of technology in transforming health education and empowerment.

## Discussion

In this research endeavor, we conducted a systematic literature review to achieve the following two objectives: (1) describe the geographic and demographic distributions of digital educational tools for SRH education for adolescents in Sub-Saharan Africa (objectives 1a and 1b) and (2) examine the types, formats, and impacts of these digital tools on target populations (objective 2). For the first objective, we identified 22 studies conducted across West, Central, East, and South Africa that focused on digital educational tools for SRH education targeting youth and adolescents aged 10 to 24 years. These studies offer valuable insights into the types of digital tools currently being employed in SRH education and highlight the regions where such tools are being most actively implemented. For the second objective, we found that a variety of digital tools, including social media, mobile apps, and gamified learning, were used to engage adolescents with accessible SRH information. These methods were generally effective, although challenges, such as the digital divide, cultural sensitivity, and the need for assessing long-term behavior change, were noted.

The 22 studies included in this review covered a broad spectrum of digital interventions, ranging from mobile apps to social media campaigns, and reflected a growing interest in digital platforms as a means of delivering SRH education to young people in Sub-Saharan Africa. The geographical distribution of the studies indicates that digital tools are being used across various cultural contexts, with notable contributions from West (eg, Nigeria and Mali), East (eg, Kenya and Tanzania), Central (eg, Cameroon and Democratic Republic of Congo), and Southern Africa (eg, Mozambique and South Africa). This regional diversity is significant as it shows that digital SRH interventions are becoming increasingly accessible across different subregions of Africa, each with unique sociocultural challenges and opportunities. Our findings suggest that digital tools can overcome geographic barriers, offering a means to engage with youth who may otherwise be excluded from formal SRH education programs due to geographical isolation, economic constraints, or cultural taboos around discussing SRH topics [41,42]. These findings are consistent with the broader literature on digital health interventions in the African region, which has highlighted the promising role of digital tools in expanding access to SRH education [31,43]. For example, in Nigeria, mobile apps focused on HIV prevention and sexual health have been shown to improve knowledge and self-efficacy among adolescents [44]. In Kenya, WhatsApp-based health campaigns have successfully increased awareness of sexual health issues among young people [45]. Conducting cross-country comparisons through systematic reviews, as performed in this work, provides valuable insights into the best practices that can be adapted and scaled across diverse African contexts. For example, WhatsApp is widely used around the world, and rather than reinventing the wheel, could developers in Nigeria adapt and implement the WhatsApp-based intervention designed for Kenyan youth by Chory et al [45] for a similar population in Nigeria? What would be the short- and long-term impacts of such an approach? Moreover, would policymakers be more inclined to support a “repackaged” intervention that is cost-effective, evidence-based, and already proven in another African context? Future research should explore these cross-country comparisons in greater depth, examining the potential for scaling successful digital interventions across African nations. By redesigning these tools to reflect the cultural, linguistic, and societal contexts of each country, researchers can assess the feasibility and cost-effectiveness of adapting existing interventions, potentially saving time and resources compared to developing entirely new solutions.

Our second objective revealed that while a variety of digital tools were used to engage adolescents with accessible SRH information, mobile apps and gamified learning emerged as particularly popular and effective approaches. Mobile apps allow adolescents to access information in a flexible, anonymous, and user-friendly format, which helps overcome the barriers posed by traditional methods of SRH education [46]. This flexibility is particularly important in contexts where discussing sexual health is stigmatized, as it empowers youth to seek information discreetly and at their own pace. Additionally, gamified learning, by integrating interactive elements, not only captures adolescents’ attention but also reinforces learning through engaging experiential methods, which may result in better knowledge retention and a deeper understanding of SRH topics [31]. Gamified learning, in particular, aligns with the preferences of a digitally native generation, where interactive and visually stimulating content is highly favored. Social media, including platforms like Facebook, WhatsApp, and Instagram, were also used to reach adolescents, leveraging peer influence and interactive communication strategies. This social aspect of learning enhances engagement by making the information feel more relevant and personal for youth. However, a major issue noted across the studies was the “digital divide,” which is used to describe the unequal access to technology and internet connectivity that can limit the effectiveness of digital SRH education programs [47]. While mobile phones are widespread in Sub-Saharan Africa, reliable internet access remains a significant barrier, particularly in rural and underdeveloped regions [45]. This digital divide may result in inequitable access to SRH resources, especially for marginalized youth [45]. Innovative solutions, such as an artificial intelligence–powered phone, have the potential to bridge the digital divide [48]. This system delivers information and advice through voice calls, eliminating the need for a smartphone or internet connection and making information accessible to individuals without reliable internet or those who cannot afford smartphones and data plans [48]. By using artificial intelligence to understand natural language queries and provide tailored information from a knowledge base, these technologies can significantly improve access to SRH education in underserved areas with limited technological infrastructure [48]. Future research should explore effective solutions to overcome the digital divide, such as offline digital tools as described, low-cost data plans, and internet infrastructure development in rural and underserved areas.

Cultural sensitivity also emerged as a critical issue. In many African contexts, cultural norms and values significantly shape how SRH topics are perceived and discussed, and failure to account for these factors can limit the effectiveness of digital SRH interventions. For instance, discussions around issues like sexual consent, gender equality, and contraception can be seen as a taboo in some regions, which may lead to resistance or disengagement from the target audience [41]. Thus, tailoring content to reflect local values, languages, and cultural norms around sexuality is critical, as failing to consider these factors could undermine the effectiveness of SRH interventions. Involving adolescents in the creation and assessment of these tools can provide essential insights into their preferences, needs, and challenges, which can significantly enhance the acceptability and effectiveness of digital interventions [49]. Moreover, including parents could create opportunities to address cultural taboos and foster more open discussions between parents and adolescents on SRH [42]. Such involvement could lead to long-term benefits, as parent-adolescent communication is a key factor in reducing adverse sexual behaviors and improving overall sexual health outcomes [42]. According to Agbeve et al [42], African parents who hold sex-positive attitudes are more inclined to engage in discussions with their adolescents that help them make informed decisions regarding their sexual health and overall well-being. When parents are actively involved in the design and implementation of digital interventions, they can provide support, guidance, and reinforcement of the messages delivered through digital platforms [50,51]. Parental involvement can help create a more comprehensive approach to sexual education, ensuring that teens not only receive the digital information but also have a trusted adult to turn to for advice and clarification [50,51]. By fostering open communication between parents and teens, digital interventions can be better tailored to the specific needs of the family, ultimately promoting healthier decision-making and outcomes for young people.

Future studies could also explore avenues for incorporating comprehensive SRH education into the formal education system, expanding the scope of topics to include healthy relationships, positive sexuality, mental health, gender equality, and consent, as was done by Fakoya et al [24]. Integrating these topics into the curriculum would not only equip adolescents with essential knowledge about SRH but also foster a more holistic understanding of well-being and personal development. By addressing issues like mental health and gender equality alongside traditional SRH topics, this approach could help challenge harmful stereotypes, promote healthier attitudes toward sexuality, and empower young people to make informed decisions, leading to positive long-term social and health outcomes.

Finally, there was a noted gap regarding the impact of these digital tools on long-term adolescent sexual behavioral change. The majority of studies measured short-term outcomes, such as knowledge acquisition, while long-term studies assessing outcomes, such as improved contraceptive use and reductions in risky sexual behavior, remained limited. The anonymity and privacy afforded by digital platforms can empower adolescents to seek information and services without fear of stigma, promoting more proactive and informed health behaviors that can be tracked over time [46]. Tracking tools can be incorporated in the design phase of the intervention development and can have input from adolescent end users. Long-term data support the sustainability of digital interventions, providing valuable information that can aid in the buy-in of investors, software developers, public health enthusiasts, and policymakers. Regardless, the implications of our findings are multifaceted and show that digital SRH tools are a promising avenue for enhancing adolescent sexual health education across the African region.

The use of a systematic literature review approach in this work has some limitations that are worth mentioning. The main limitations of our work are related to the methodology. There is a risk of selection bias, meaning that some relevant studies may have been omitted or that the included studies may not fully represent all available research on the topic. However, we believe that our extensive use of multiple databases (Ovid-MEDLINE, Google Scholar, PubMed, and ERIC) mitigated the risk of selection bias inherent in systematic reviews. A meta-analysis could further inform the selection bias risk and the heterogeneity of the included studies, and quantify the associations reported.

In conclusion, although digital SRH tools are a promising avenue for enhancing adolescent sexual health education in Sub-Saharan Africa, there is a need for infrastructure improvements to ensure the tools are equitably accessible and a need for understanding long-term impacts and sustainability. Policymakers, educators, and public health researchers must collaborate to ensure that digital innovations in SRH education are grounded in evidence, culturally sensitive, and widely accessible to all adolescents, regardless of socioeconomic status or geographical location. This will address the sustainable development goals of reducing the global disparities in SRH health (goal 10) and achieving good health and well-being across nations (goal 3).

## Appendix

Multimedia Appendix 1 Line-by-line search to retrieve titles and abstracts.

Multimedia Appendix 2 PRISMA (Preferred Reporting Items for Systematic Reviews and Meta-Analyses) checklist.

## Abbreviations

HCD: human-centered design
LMIC: low- and middle-income country
SRH: sexual and reproductive health
STI: sexually transmitted infection

## Data Availability

All processes are outlined in the Methods section. Additional information beyond what is reported in the manuscript (eg, template data collection forms, data extracted from the included studies, data used for all analyses, analytic codes, etc) is available from the corresponding author upon reasonable request.
