# Supplementary material for: Exploring the Use of Digital Educational Tools for Sexual and Reproductive Health in Sub-Saharan Africa: Systematic Review
Source: JMIR Public Health Surveill. 2025 Feb 26;11:e63309. doi: 10.2196/63309 (PMC11904370; doi:10.2196/63309)
Supplement: Multimedia Appendix 1 [file publichealth_v11i1e63309_app1.docx]

**Multimedia Appendix 1.** Line-by-line search to retrieve titles and abstracts.

| Line Number | Search String |
| --- | --- |
| Line 1 | Adolescent/ |
| Line 2 | (adolescent* or teen* or youth).ti,ab,kf. |
| Line 3 | 1 or 2 |
| Line 4 | digital health education/ or health promotion/ or sex education/ |
| Line 5 | ((health or reproduction or reproductive or sex or sexual) adj2 (education or promotion or resources or training)).ti,ab,kf |
| Line 6 | 4 or 5 |
| Line 7 | 3 and 6 |
| Line 8 | hiv infections/ or acquired immunodeficiency syndrome/ or sexually transmitted diseases/ or exp sexually transmitted diseases, bacterial/ or chancroid/ or chlamydia infections/ or gonorrhea/ or syphilis/ or sexually transmitted diseases, viral/ or herpes genitalis/ or papillomavirus infections/ |
| Line 9 | (aids or hiv or (sexually adj3 transmit*) or chlamydia or gonorrhea or herpes or HPV or papillomavirus or syphilis).ti,ab,kf. |
| Line 10 | Contraception/ |
| Line 11 | contraceptive agents/ or exp contraceptives, oral/ |
| Line 12 | reproductive behavior/ or contraception behavior/ |
| Line 13 | Safe Sex/ or Condoms/ |
| Line 14 | ((birth adj control) or condom or condoms or contracepti* or (safe adj sex)).ti,ab,kf. |
| Line 15 | 8 or 9 or 10 or 11 or 12 or 14 |
| Line 16 | 7 and 15 |
| Line 17 | exp africa/ |
| Line 18 | (Africa or Angola or Benin or Botswana or Burkina Faso or Burundi or Cabo Verde or Cameroon or Central African Republic or Chad or Comoros or Congo or Cote d'Ivoire or Djibouti or Eritrea or Eswatini or Ethiopia or Gabon or Gambia or Ghana or Accra or Kumasi or Tamale or Sekondi-Takoradi or Guinea or Kenya or Lesotho or Liberia or Madagascar or Malawi or Mali or Mauritania or Mozambique or Namibia or Niger or Nigeria or Rwanda or Sao Tome or Principe or Senegal or Seychelles or Sierra Leone or Somalia or Sudan or Tanzania or Togo or Uganda or Zambia or Zimbabwe).ti,ab,kf. |
| Line 19 | 17 or 18 |
| Line 20 | 16 and 19 |
| Line 21 | "systematic review".pt. or "Systematic Reviews as Topic"/ or "Cochrane Database of Systematic Reviews".jn. or (evidence report technology assessment or evidence report technology assessment summary).jn. or (((comprehensive* or integrative or mapping or rapid or realist or scoping or systematic or systematical or systematically or systematicaly or systematicly or umbrella) adj3 (bibliographical or bibliographically or bibliographics or literature or review or reviews)) or (state adj3 art adj1 review) or (research adj2 synthesis) or ((data or information) adj3 synthesis)).ti,ab,kf. or ((data adj2 (extract or extracting or extractings or extraction or extraction)).ti,ab,kf. and ("review".ti. or "review".pt.)) or (((electronic or searched) adj2 database*) and (eligibility or excluded or exclusion or included or inclusion)).ti,ab,kf. or (overview adj4 reviews).ti,ab,kf. or ((review adj3 (rationale or evidence)).ti,ab. and "review".pt.) or (PRISMA or (preferred adj1 reporting)).ab. or (cinahl or (cochrane adj3 (trial or trials)) or embase or medline or psyclit or (psycinfo not (psycinfo adj1 database)) or pubmed or scopus or (sociological adj1 abstracts) or (web adj2 science)).ab. |
| Line 22 | 20 and 21 |
| Line 23 | 16 and 21 |
| Line 24 | 23 not 22 |
| Line 25 | (20 not 22) and english.la. |
| Line 26 | (ghana/ or (Ghana or Accra or Kumasi or Tamale or Sekondi-Takoradi).ti,ab,kf.) and 25 |
| Line 27 | 25 not 26 |
| Line 28 | limit 27 to yr="2013 - 2024" |
| Line 29 | limit 27 to yr="2003 - 2012" |
| Line 30 | limit 27 to yr="1993 - 2002" |
